# Stark control of electrons along nanojunctions

**DOI:** 10.1038/s41467-018-04393-4

**Published:** 2018-05-25

**Authors:** Liping Chen, Yu Zhang, GuanHua Chen, Ignacio Franco

**Affiliations:** 1 Department of Chemistry, University of Rochester, Rochester, NY 14627 USA; 2 Department of Chemistry, The University of Hong Kong, Pokfulam Road, Hong Kong SAR, China; 3 Department of Physics, University of Rochester, Rochester, NY 14627 USA; 4 Present Address: Theoretical Division, Los Alamos National Laboratory, Los Alamos, NM 87545 USA

## Abstract

Ultrafast control of currents on the nanoscale is essential for future innovations in nanoelectronics. Recently it was experimentally demonstrated that strong non-resonant few-cycle 4 fs laser pulses can be used to induce phase-controllable currents along gold–silica–gold nanojunctions in the absence of a bias voltage. However, since the effect depends on a highly non-equilibrium state of matter, its microscopic origin is unclear and the subject of recent controversy. Here we present atomistically detailed (time-dependent non-equilibrium Green’s function) electronic transport simulations that recover the main experimental observations and offer a simple intuitive picture of the effect. The photoinduced currents are seen to arise due to a difference in effective silica-metal coupling for negative and positive field amplitudes induced by lasers with low temporal symmetry. These insights can be employed to interpret related experiments, and advance our ability to control electrons in matter using lasers.

## Abstract (web summary)

Strong non-resonant few-cycle laser pulses can be used to induce ultrafast phase-controllable currents along nanojunctions but the microscopic origin is unclear. Here, the authors present time-dependent quantum transport simulations that recover the experimental observations and offer an intuitive picture of the effect.

## Introduction

A general goal in our quest to control matter and energy is the design of strategies to control electronic properties and electron dynamics using coherent laser sources^1–3^. In addition to its interest at a fundamental level, lasers permit manipulation on an ultrafast timescale opening the way to control the ability of matter to chemically react, conduct charge, absorb light, or other properties, in a femto to attosecond timescale.

In a recent experiment, Schiffrin et al.^4^ demonstrated how a strong (*I* ~ 10^13^–10^14^ W cm^−2^) non-resonant few-cycle 4 fs laser pulse can be used to induce currents along gold–silica–gold nanojunctions in the absence of a bias voltage. Phenomenologically, these currents arise due to the nonlinear interaction of the junction with a laser pulse that has a low temporal symmetry^5^. By varying the carrier envelope phase it is possible to vary the degree of time asymmetry of the incident radiation, and thus the direction and magnitude of the photoinduced current.

The experiment marks a new frontier in laser control of electronic dynamics and is the fastest existing method for the generation of currents. However, the microscopic origin of this rather spectacular effect is unclear and the subject of recent controversy. This is because the applied electric field is just below the dielectric breakdown of the silica, and the laser-frequencies are far detuned from the electronic transitions in the system such that Stark effects, the shifts in energy levels due to application of an electric field, can play an important role.

In a broader context, this experiment falls into a general class of symmetry breaking laser-control scenarios known to induce phase-controllable currents in the absence of a bias voltage^1,6–9^. In the traditional version of this laser control, laser pulses *E*(*t*) = $\epsilon _\omega$ cos(*ωt* + *ϕ*_*ω*_) + $\epsilon _{2\omega }$ cos(2*ωt* + *ϕ*_2*ω*_) with frequency components *ω* and 2*ω* are used to photoexcite a spatially symmetric system from a bound state to a given energy in the continuum by means of a near resonance one-photon and two-photon excitation. Since odd-photon processes connect states with opposite parity while even-photon process connect states with the same parity, simultaneous photoexcitation via a one- and two-photon process creates a state in the continuum of no definitive parity. This breaks the spatial symmetry of the system and generates a net phase-controllable current $I\sim \overline {E(t)^3}$ ~ $\epsilon _\omega ^2\epsilon _{2\omega }{\kern 1pt} {\mathrm{cos}}\left( {2\phi _\omega - \phi _{2\omega }} \right)$, where the overbar denotes time averaging. More generally, the scenario applies under resonant and non-resonant condition by using lasers, such as those employed in the experiment, that are neither symmetric nor antisymmetric with respect to sometime *t*′ [i.e., *E*(*t* − *t*′) ≠ *E*(−(*t* − *t*′)) and *E*(*t* − *t*′) ≠ −*E*(−(*t* − *t*′))]^5^. The nonlinear response of matter mixes the frequencies and harmonics of the laser field and leads to the generation of a zero-frequency DC component in the current *I*. Such symmetry breaking component arises due to excitation via an even *n* and odd *m* photon process, appears in the $I\sim \left| E \right|^{n + m}$ order in the response^1,10^, and is often referred to as *n* vs. *m* multiphoton interference effect.

The experiments are performed by shining a strong 4 fs few-cycle laser pulse of varying amplitude (0.4–1.7 V Å^−1^) and fixed carrier envelope phase *φ* to a 50 nm metal-fused silica-metal junction. The laser polarization is chosen to be along the junction direction, there is no bias voltage across the junction, and the laser central frequency (*ħω* = 1.7 eV) is far detuned from the electronic transitions across the 9 eV gap of the silica. The laser irradiates both the metal and the silica, and any response that is not dependent of the carrier envelope phase is experimentally eliminated.

Currently, there are four theories that seek to explain the microscopic origin of the effect. The mechanism proposed in refs. ^4,11^ is based on Zener band-to-band tunneling^12,13^ induced by an electric field and a theory for metallization of dielectrics through Stark shifts^14^. This mechanism is also used to interpret the simulations in ref. ^15^. In turn, ref. ^16^ adopts a more traditional perspective and argues that the spectral bandwidth of the pulse can sustain a resonant 5 vs. 6 photon absorption process that creates real carriers and induces symmetry breaking. A third interpretation^17^ is based on the idea of creating virtual carriers in the conduction and valence bands through non-resonant 1 vs. 2 and 2 vs. 3 multiphoton quantum interference. The possibility of generating virtual carriers through Stark effects was also suggested in ref. ^11^.

A fourth possibility arises from an early proposal^18^ to induce currents in nanojunctions through Stark shifts. The basic idea is to use a field with a low temporal symmetry with frequencies far detuned from electronic transitions in the photoactive material such that Stark effects, and not near-resonance photon absorption, dominate the dynamics. Through changes in the metal-semiconductor band alignment via Stark shifts, the time asymmetry of the pulse leads to a difference in effective metal-semiconductor coupling for positive and negative laser field amplitude, ultimately leading to phase-controllable currents.

While the experiments are performed in a nanojunction, the proposals in refs. ^4,11,15–17^. consider the silica as a periodic solid and do not take into account any effects that the gold–silica interface may play in the production of currents. Thus, even if virtual or real carriers are created through Stark or multiphoton interference, it is unclear if these carriers can be collected by the metallic contacts to form a measurable current. In turn, ref. ^18^, while it does take into account the interface, does not consider that the electric field of light also induces an AC bias voltage across the junction (a basic feature recognized by Tien and Gordon^19^ that leads to photon-assisted tunneling). Further, simulations in ref. ^18^ were performed using a 1.3 eV photoactive material using much weaker ~10^9^ W cm^−2^ lasers and, as a consequence, it is not clear if the identified mechanism is at play in the experiments.

What is then the microscopic origin of the experimental observations?

In this paper, we present state-of-the-art atomistic simulations of the laser-induced time-dependent electronic transport along gold–silica–gold nanojunctions that recover the experimental observations and offer a simple intuitive picture for the origin of the effect. The simulations explicitly take into account the nanoscale nature of the experimental setup and the crucial role of the silica–gold interface in the current rectification. This contrasts with previous efforts^4,15–17,20^ that model the silica as bulk matter. Using them we assess the feasibility of mechanisms that have been proposed^4,11,15–18,20^ to explain the observed currents.

## Results

### Computational approach

The simulations are based on solving the single-particle Liouville von Neumann equation for the nanojunction in the presence of radiation using a non-equilibrium Green’s function method^21^ (TD-NEGF) for time-dependent electronic transport in the wide band limit. While the experiments are performed on fused silica, the simulations employ a quantitative atomistic model of *α*-quartz that is developed from density functional theory (DFT). Since the symmetry breaking effect occurs along the direction of laser polarization, we focus on quasi one-dimensional model junctions of varying length as they capture the essential physics of the effect. Specifically, we consider one-dimensional slabs of *N* unit cells of *α*-quartz along a given crystallographic direction $\widehat {\boldsymbol{d}}$ (*a*, *b*, or *c*) connected by its ends to macroscopic metallic contacts. We focus on the *a* (*E* || *a*) and *c* (*E* || *c*) directions since the *b* direction is equivalent to *a*.

**Fig. 1:**
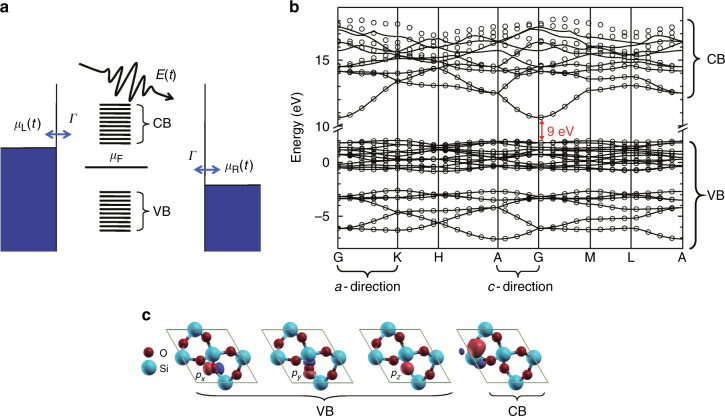
Electronic structure of the gold–silica–gold nanojunctions. **a** Energy diagram of the nanojunction in the presence of a laser *E*(*t*). The junctions are composed of *N* unit cells of *α*-SiO_2_ grown along the *a* (*E* || *a*) or *c* (*E* || *c*) crystallographic directions and connected by its ends to metallic contacts. The lead’s Fermi energy *μ*_F_ is taken to be between the valence band (VB) and conduction band (CB). Here, *Γ*/*ħ* is the charge transfer rate between each Wannier functions at the terminal unit cells to the corresponding lead and *μ*_*α*_(*t*) the chemical potential of left (*α* = L) and right (*α* = R) contact. **b** Band structure of *α*-SiO_2_ computed using density functional theory (solid lines) and the generalized tight-binding model (open circles) based on maximally localized Wannier functions (MLWFs). The model consists of 27 MLWFs per unit cell that result in 18 VBs and 9 CBs. It accurately reproduces the band structure in a wide energy range and the 9 eV experimental band gap. The relevant electronic structure along the *a* and *c*-direction are highlighted. **c** Isosurface contours of four representative MLWFs (red for positive value and blue for negative)

A schematic representation of the metal-silica-metal nanojunction is shown in Fig. 1a. The Wannier functions in the terminal unit cell are assumed to couple identically to the metallic contact at their terminal end. The quantity *ħ*/*Γ* determines the rate of charge exchange between the silica and contacts and is fixed at a model but realistic value of *Γ* = 0.1 eV. The Fermi energy *μ*_F_ of the gold contacts is taken to be in the band gap and its exact value is a modeling parameter. The laser-matter interactions is considered in dipole approximation and the metallic contacts are assumed to behave like perfect metals that completely reflect the incident radiation. The vector potential *A*(*t*) associated with the electric field $E(t) = - \dot A(t)$ employed in the simulations is of the form *A*(*t*) = (*E*_0_/*ω*) exp[−(*t* − *t*_c_)^2^/(2*σ*^2^)] sin(*ω*(*t* − *t*_c_) + *φ*). This form guarantees that the few-cycle *E*(*t*) of amplitude *E*_0_ is an AC source, as ${\int}_{ - \infty }^\infty {\kern 1pt} E(t){\mathrm{d}}t$ = *A*(−∞) − *A*(∞) = 0. To mimic the experiments, we choose a laser pulse centered at *t*_c_ = 0 fs of width *σ* = 2 fs and central frequency *ħω* = 1.7 eV. The carrier envelope phase *φ* is the main source of control of the electronic currents in the experiment. In the model, the effect of *E*(*t*) on the metallic contacts is to setup a total potential drop across the junction *V*(*t*) = *eDE*(*t*), where *D* is the total length of the junction and *e* the electronic charge, by rigidly shifting the energy levels of left and right contacts^19^. In the wide band limit, this shift is computationally captured by a time-dependence of the chemical potentials of the left *μ*_L_(*t*) = *μ*_F_ − $\frac{1}{2}eDE(t)$ and right *μ*_R_(*t*) = *μ*_F_ + $\frac{1}{2}eDE(t)$ contacts.

The net current passing through the nanojunction is calculated as the average current flowing into the two leads *I*(*t*) = (*I*_L_(*t*) − *I*_R_(*t*))/2, where *I*_*α*_(*t*) is the current entering lead *α*. The total charge transferred between two leads at time *t* is given by *Q*(*t*) = ${\int}_{ - \infty }^t {\kern 1pt} I(t{\prime}){\rm d}t{\prime}$, while the accumulated charge in the silica region is *Q*_acc_(*t*) = ${\int}_{ - \infty }^t {( I_{\mathrm{L}}(t{\prime}) + I_{\mathrm{R}}(t{\prime})}){\kern 1pt} {\rm d}t{\prime}$.

### Accurate generalized tight-binding model for the silica

To be able to describe the dynamics of a junction with hundreds of atoms driven by strong laser fields, we developed an accurate and computationally efficient generalized tight-binding model (GTB) of the silica from first principle computations. For this, we computed the Bloch states and band structure of *α*-quartz using DFT (modified Becke-Johnson meta-GGA functional^22^), and used the results to generate an orthonormal basis of maximally localized Wannier functions (MLWFs) via unitary transformation^23^. The matrix elements between these Wannier functions are then employed to build a Hamiltonian for the silica and its interaction with the laser. The resulting basis consist of 27 MLWFs per unit cell, that capture 18 Valence Bands (VB) and 9 Conduction Bands (CB). Figure 1b shows the resulting ground-state band structure of *α*-SiO_2_^24^ (*a* = *b* = 4.9137 Å, *c* = 5.4047 Å, *α* = *β* = 90°, and *γ* = 120°) computed with DFT (solid lines) and the GTB model (open circles). Figure 1c shows the isosurface contours of four representative MLWFs in *α*-SiO_2_ (red for positive value and blue for negative). The MLWFs that compose the VB correspond to the *p*_*x*_, *p*_*y*_, and *p*_*z*_ orbitals of 6 O atoms, while the CB MLWFs involve contributions from both Si and O atoms.

As shown in Fig. 1b, the GTB accurately reproduces the first-principle-based band structure in a wide energy range and the bulk 9 eV band gap. The one-dimensional model slabs employed in the transport simulations have a 16–18% larger band gap [10.4 eV (*E* || *a*) and 10.6 eV (*E* || *c*)] because they neglect tight-binding couplings in directions perpendicular to the junction. The GTB has no adjustable parameters and retains the atomistic detail of first principle approaches. In addition, it allows one to select the number and type of bands that participate in the dynamics. In this way, it offers a powerful theoretical tool to interpret the experiments. Additional details of the simulation approach are included in the Methods section.

### Phase and size dependence of the photocurrents

**Fig. 2:**
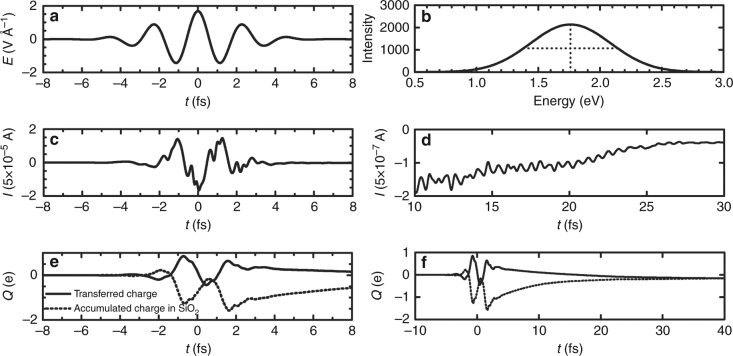
Time dependence of the laser-induced currents. **a**–**d** Time-dependent currents *I*(*t*) during (**c**) and after (**d**) photoexcitation by the non-resonant 4 fs laser pulse *E*(*t*) with maximum amplitude 1.7 V Å^−1^ and *φ* = *π* shown in **a** (*N* = 6, *E* || *a*, *μ*_F_ = 7.5 eV). The Fourier transform of *E*(*t*) quantifying its spectral content is shown in **b**. **e**, **f** Transferred charge (solid line) through the junction and accumulated charge (dashed line) in the SiO_2_ during (**e**) and after (**f**) the pulse

Figure 2 shows the currents induced by the 4 fs laser pulse on a *N* = 6 junction (*E* || *a*). The laser (Fig. 2a, b) transiently generates large currents (Fig. 2c, d). Figure 2e, f shows the net charge transferred across the junction (solid line) and the accumulated charge in the silica (dashed line). As shown, the laser photoejects electrons and leaves the silica charged (Fig. 2e). Only a fraction of the photoejected electrons form part of the net current. After the laser, the metallic contacts inject charge back into the system and restore charge neutrality in 20–30 fs (Fig. 2f).

**Fig. 3:**
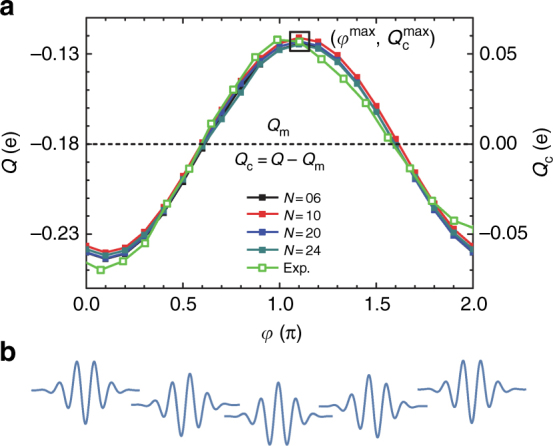
Phase and size dependence of the laser-induced currents. **a** Dependence of the net transferred charge on the laser carrier envelope phase *φ* and junction size *N* (*E* || *a*, *μ*_F_ = 7.5 eV, *E*_0_ = 1.7 V Å^−1^). **b** Scheme of the laser field for different *φ*. In agreement with experiment^4^ (green, magnitude scaled), *Q*(*φ*) is a sinusoidal function of *φ* with a period of 2*π*. The total response *Q* = *Q*_m_ + *Q*_c_ consist of a phase-controllable component *Q*_c_ and an uncontrollable one *Q*_m_ = $\frac{1}{{2\pi }}{\int}_0^{2\pi } {\kern 1pt} Q(\varphi ){\mathrm{d}}\varphi$ that arises due to the spatial asymmetry in *α*-quartz. The black square signals the maximum transferred charge $Q_{\mathrm{c}}^{{\mathrm{max}}}$ and *φ*^max^. Note that the effect is essentially independent of junction size

Figure 3a shows the dependence of the net charge extracted *Q* = *Q*_m_ + *Q*_c_ after the system has equilibrated on the carrier envelope phase *φ*. The effect of *φ* on the laser pulse is shown in Fig. 3b. There are two components to the response: a component *Q*_m_ that is independent of *φ* that arises because of the inherent spatial asymmetry of *α*-SiO_2_, and a phase-controllable component *Q*_c_. The experiments are designed to only capture *Q*_c_. The simulations capture the experimentally observed sinusoidal dependence of the magnitude and sign of *Q*_c_ on *φ*. The slight discrepancy in the control map between theory and experiment arises because the experiment exhibits dispersion effects as *φ* is varied that are not included in the simulations. Importantly, the simulations show that the effect is largely independent on junction size *N*, as the net extracted charge observes essentially no dependence with the number of unit cells for *N* = 6, 10, 20, 24. This observation is consistent with experiments performed with 50 and 500 nm junctions in wedged and flat geometries, respectively, that suggest a mild dependence of the effect on junction size (Fig. S8 in ref. ^4^). Below, we focus on *N* = 6 as it describes well the behavior of the longer *N* = 24 junction and is expected to be representative of the *N* ~ 100 experimental setup.

### Dependence of the photocurrents on laser intensity

**Fig. 4:**
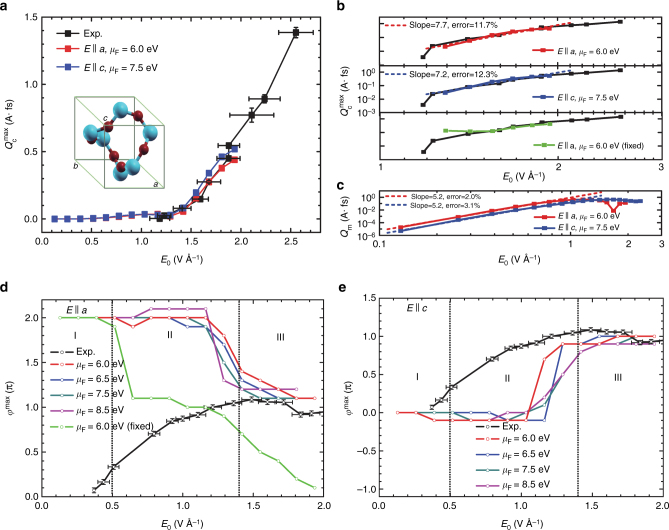
Dependence of the maximum transferred charge $Q_{\mathrm{c}}^{{\mathrm{max}}}$ and the carrier envelope phase *φ*^max^ required to induce it on the laser amplitude *E*_0_. **a** Controllable $Q_{\mathrm{c}}^{{\mathrm{max}}}$ component of the response as a function of maximum laser amplitude for *N* = 6 junctions constructed along the *a* (red) and *c* (blue) direction. The crystal structure of *α*-SiO_2_ depicting the crystallographic directions is shown in the inset. To compare with experimental magnitudes^4^ (black), simulation results are scaled by a cross-section *η* that accounts for the effective area of illumination by the laser pulse and differences in the optical response between fused silica and particular directions in alpha quartz [*η* = 3.571 × 10^5^ unit cells (9.484 × 10^−2^ μm^2^) in the *bc* plane for *E* || *a*, and *η* = 2.632 × 10^5^ unit cells (5.503 × 10^−2^ μm^2^) in *ab* for *E* || *c*.]. **b** Log–log plots quantifying the power dependence of the simulated effect on the laser amplitude $Q_{\mathrm{c}}^{{\mathrm{max}}} \propto \left| {E_0} \right|^r$ as determined by the slopes. For comparison purposes, simulations using fixed, as opposed to time-dependent, chemical potentials are shown in green. **c** Power dependence of the uncontrollable component of the response *Q*_m_. Simulations recover the experimental intensity dependence of the effect ($Q_{\mathrm{c}}^{{\mathrm{max}}} \propto \left| {E_0} \right|^7$) and show that $Q_{\mathrm{m}} \propto \left| {E_0} \right|^5$. **d**, **e** Dependence of the carrier envelope phase required for maximum charge transfer *φ*^max^ (cf. Fig. 3a) on the laser amplitude for **d** *E* || *a* and **e** *E* || *c*. The experimental results^20^ (black) are also included for comparison. Simulations agree with experiment in region III where appreciable currents are observed. Simulation results using a fixed chemical potential (in green) do not agree with experiments. Throughout, error bars show the experimental standard deviation

Figure 4 shows a comparison between experimental and computational maximum extracted charge $Q_{\mathrm{c}}^{{\mathrm{max}}}$ and *φ*^max^ as a function of laser amplitude *E*_0_. For a given laser amplitude, $Q_{\mathrm{c}}^{{\mathrm{max}}}$ is extracted by scanning the dependence of the extracted charge *Q*_c_ on *φ* and recording the maximum charge $Q_{\mathrm{c}}^{{\mathrm{max}}}$ in the control map. To compare with experiments, the simulation results are scaled by a factor *η* which represents the illumination cross-section area which is an experimentally unknown parameter. Simulations correctly capture the intensity dependence of the effect up to a laser amplitude of 2 V Å^−1^. To capture observations beyond this laser amplitude, the generalized tight-binding model would require a larger number of bands. Note that the intensity and phase dependence of the control is approximately independent of the crystallographic direction in the model junction. This insensitivity makes the model of *α*-quartz useful in the description of the experiment.

**Fig. 5:**
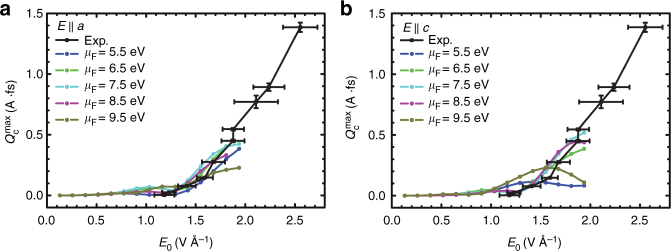
Dependence of the net transferred charge on the position of the Fermi energy. *N* = 6 junctions are constructed along the **a** *a*-direction and **b** *c*-direction. Experimental values and error bars (standard deviation) are taken from ref. ^4^. Note that the results are insensitive to the position of the Fermi energy, except when *μ*_F_ is chosen to be close to the top of the VB (as in *μ*_F_ = 5.5 eV) or to the bottom of the CB (as in *μ*_F_ = 9.5 eV) (cf. Fig. 1b)

An unknown variable in the simulations is the position of the Gold’s Fermi energy *μ*_F_ with respect to the silica. To test the dependence of the results on *μ*_F_, this parameter was varied between the top of the VB (1.8 eV) and the bottom of the CB (10.7 eV). As shown in Fig. 5, the results are approximately independent of *μ*_F_ under a wide range of values. Disagreement with experiment starts to emerge when the *μ*_F_ is chosen to be close to the band edges.

To examine possible effects of the 16–18% overestimation of the energy gap *E*_g_ by the model nanowires, we performed simulations with a larger central frequency *ω* such that the experimental *E*_g_/*ħω* = 5.3 ratio is maintained. As shown in Supplementary Fig. 1, increasing *ω* changes the illumination cross-section *η* but leaves the qualitative features of the control map unchanged.

As yet another point of contact with experiment^20^, we examined the dependence of the carrier envelope phase required to achieve the maximum charge *φ*^max^ on the laser amplitude. Figure 4 shows such a dependence when the junction is constructed along the *a* (Fig. 4d) and *c* (Fig. 4e) crystallographic directions. For weak fields (region I) the simulations recover the experimentally observed *φ*^max^ ≈ 0 phase required for maximum current. For intermediate intensities (region II), we observe that *φ*^max^ is sensitive to the particular crystallographic direction. Since the experiments are performed in fused silica, while the simulations are done in *α*-quartz, the disagreement between theory and experiment arises because the microscopic model in the simulations does not coincide exactly with the material employed in the experiment. Importantly, in the most relevant region where appreciable currents are observed ($\left| {E_0} \right| > 1.4$ V Å^−1^, region III), the simulations recover the experimentally observed *φ*^max^ ≈ *π* and the independence of *φ*^max^ on the laser intensity^20^.

### Microscopic origin of the effect

As shown above, the simulations recover the main experimental observations including the phase dependence, intensity dependence and size independence of the effect. We are now thus in a position to examine the microscopic origin of the effect, and the relevance of previously proposed mechanisms.

### Importance of the 5 vs. 6 photon absorption coherent control scenario

**Fig. 6:**
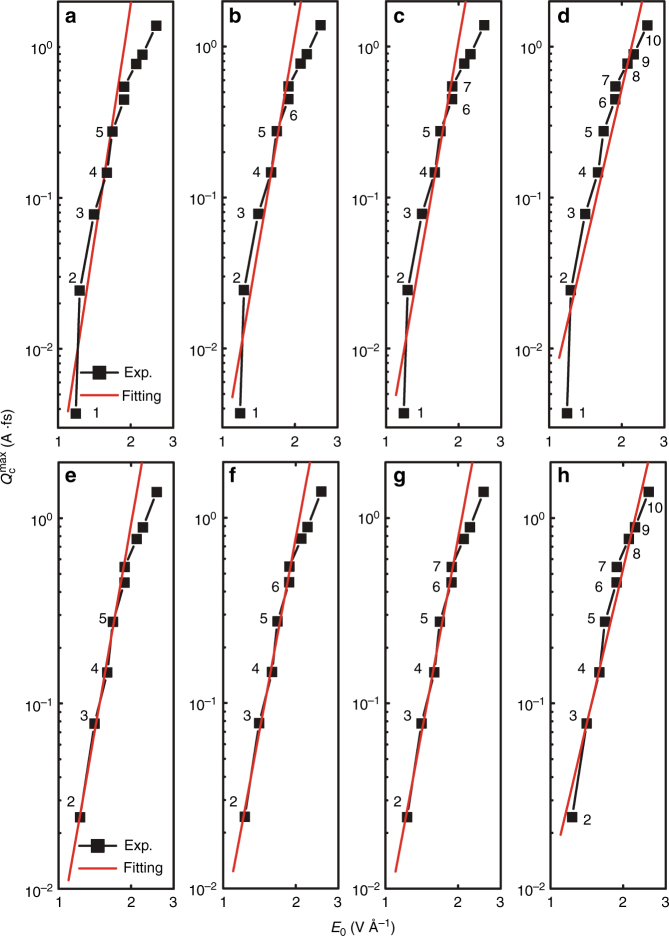
Scaling of the experimental maximum transferred charge $Q_{\mathrm{c}}^{{\mathrm{max}}} \propto \left| {E_0} \right|^r$ with applied laser field amplitude. The different log–log plots correspond to different fits that employ the sets of consecutive data points labeled in each panel. Different sets can capture different regimes in the laser matter interaction. The resulting power scaling *r*, quality of the least-square fitting *R*^2^ ≤ 1 and error Δ $[r , \,R^2\,({\Delta })]$ are as follows: **a** [10.4, 0.84 (21.2%)], **b** [9.2, 0.87 (17.3%)], **c** [9.0, 0.89 (13.9%)], **d** [6.9, 0.88 (12.4%)], **e** [7.4, 0.98 (8.5%)], **f** [6.9, 0.98 (6.9%)], **g** [7.0, 0.99 (5.3%)], **h** [5.5, 0.95 (8.1%)]. Data points 2–7 (**g**) correspond to a regime that is well characterized by a given order in the perturbative response of the system to the electric field, and yield *r* = 7 (*R*^2^ = 0.99, *Δ* = 5.3%)

To test this possible mechanism^16^, we examined the experimental power *r* dependence of the effect on the electric field amplitude $Q_{\mathrm{c}}^{{\mathrm{max}}}\sim \left| {E_0} \right|^r$. The 5 vs. 6 scenario should exhibit an $\left| {E_0} \right|^{11}$ dependence. Figure 6 shows fits of the experimental data to $Q_{\mathrm{c}}^{{\mathrm{max}}}\sim \left| {E_0} \right|^r$ that take into account different sets of consecutive experimental points. The regime of the response that can be captured by a single power law (points 2–7, *R*^2^ = 0.99, error 5.3%) offer an *r* = 7 which is inconsistent with a 5 vs. 6 scenario. Only the fit with points 1–5 offer an *r* = 10.4 consistent with a 5 vs. 6 scenario and that fit is, statistically, a poorer representation of the data (*R*^2^ = 0.84, error 21.2%). In the simulations *r* ≈ 7 (Fig. 4b) in agreement with experiments.

Further note that a resonant 5 vs. 6 scenario is expected to exhibit a strong dependence on the length of the material *N* because the magnitude of the transition dipoles between energy eigenstates (and the number of available transitions) increase with *N*. Such length dependence is not observed in experiments nor simulations (cf. Fig. 3a and Fig. S8 in ref. ^4^).

These experimental and numerical observations suggest that 5 vs. 6 control is not the dominant mechanism underlying the effect.

### Importance of Stark effects

An additional aspect that requires clarification is whether the laser-induced dynamics is due to near-resonance multiphoton absorption or due to Stark shifts. While the central frequency of the pulse is far detuned from the band-gap, the laser is intense enough that competition between these two effects is possible. This distinction is important because multiphoton excitation will generate real charge carriers, while Stark shifts will reversibly deform the electronic structure of the material generating vastly different mechanisms for the response.

To address this, in Fig. 4c we examine the power dependence of the uncontrollable part of the response, $Q_{\mathrm{m}}\sim \left| {E_0} \right|^r$. To photoexcite electrons across the energy gap, 4–6 photons from the pulse need to be absorbed which implies that 8 ≤ *r* ≤ 12 if multiphoton absorption plays a role. As shown in Fig. 4c for laser amplitudes $\left| {E_0} \right|$ < 1 V Å^−1^, *Q*_m_ scales with *r* ≈ 5 and then saturates, which is a power dependence that is considerably below the threshold for multiphoton absorption. We thus conclude that Stark effects due to non-resonant laser-matter interactions dominate the dynamics.

### Importance of Wannier-Stark metallization

**Fig. 7:**
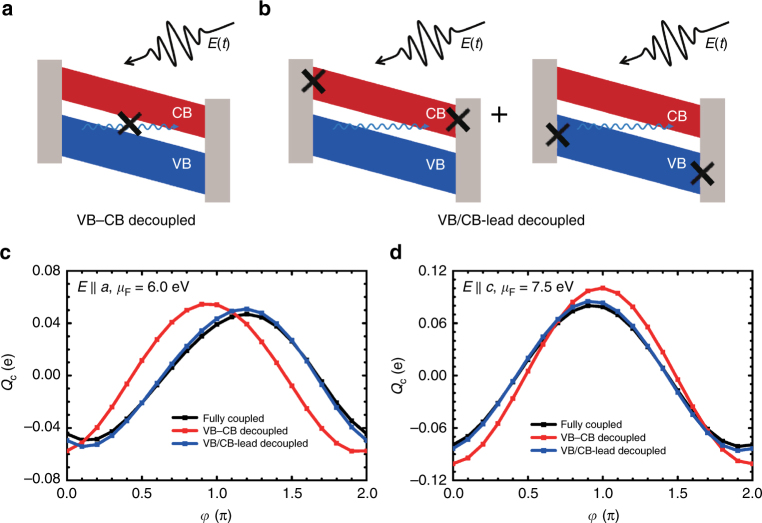
Importance of Zener tunneling on the photoinduced currents. **a**, **b** Scheme of the junction in the presence of an electric field. The wiggly line represents Zener tunneling between the VB and CB. To test its importance two simulation conditions are considered: **a** Dynamics with Zener tunneling between VB and CB removed (VB-CB decoupled); and **b** Dynamics obtained by independent electron and hole channels (VB/CB-lead decoupled). The latter is obtained by performing simulations in which only the VB (hole transport) or the CB (electron transport) are connected to the leads and adding their contributions to the total current, as indicated in **b**. **c**, **d** Control map for a *N* = 6 junction in the two cases depicted in the upper panel for junctions constructed along the **c** *a*-direction and **d** *c*-direction. The full simulations results are shown in black. Partial or complete suppression of Zener tunneling pathways does not have a major effect on the photoinduced currents

In refs. ^4,11,15^ the effect is interpreted through Wannier-Stark metallization effects that require Zener tunneling to emerge. To test this interpretation, we performed numerical experiments (Fig. 7) in which Zener tunneling pathways are eliminated completely or partially from the dynamics. Specifically, we examined the control map (*E*_0_ = 1.7 V Å^−1^) under circumstances in which the CB and VB are completely decoupled from one another (Fig. 7a, red line in c and d) eliminating Zener tunneling effects from the dynamics. This is achieved by setting the Hamiltonian matrix elements between the Wannier basis states that form the VB and those that form the CB to zero. We also examined a case (Fig. 7b, blue line in c and d) where Zener tunneling is maintained but transport is assumed to go through the VB (hole transport) or the CB (electron transport) independently, with no transport pathways that involve both bands as required for mechanisms based on Zener tunneling. This is done by performing two separate simulations in which either the VB or the CB is disconnected from the leads and adding their two separate contributions to the current. If Zener tunneling is an essential component of the dynamics, case (i) should exhibit no net currents while case (ii) should exhibit suppressed currents.

As shown in Fig. 7, eliminating completely Zener tunneling shifts slightly (by ~ 0.25*π*) the control map but has no appreciable incidence on the magnitude of the effect. Similarly, considering that transport does not involve pathways that involve both bands has a minor impact on the control map. We are thus forced to conclude that Zener tunneling is not essential for the description of the experimental observations and, thus, that Wannier-Stark metallization^14^ does not underlie the effect.

The relevance of the mechanism proposed in ref. ^18^ is discussed below.

### Instantaneous level alignment as an interpretative tool

**Fig. 8:**
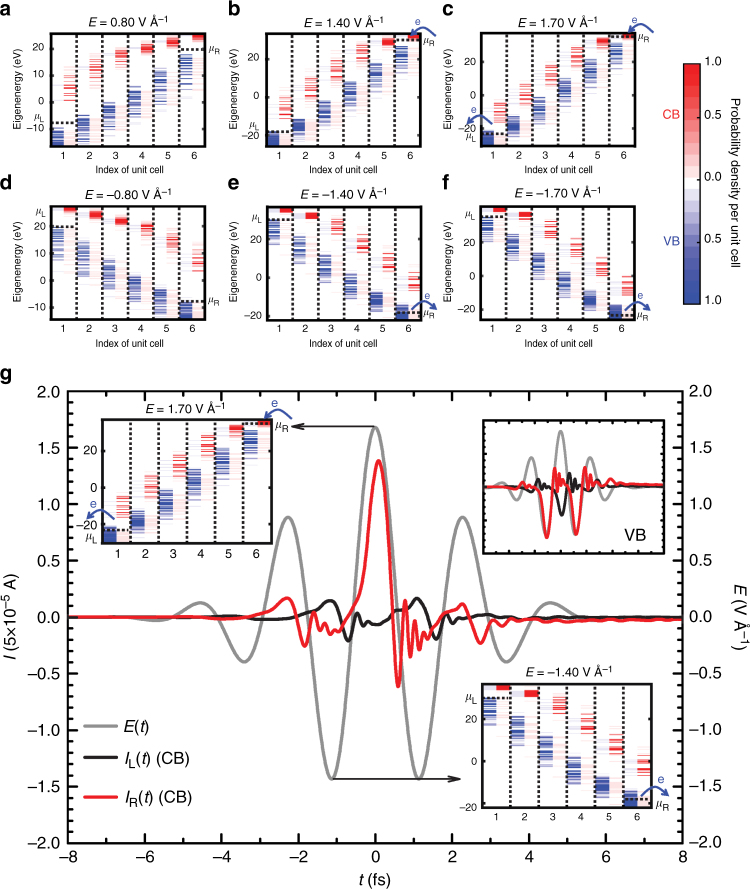
Mechanism underlying the laser-induced currents. **a**–**f** Laser-dressed single-particle eigenstates of SiO_2_ (*N* = 6, *E* || *a*, *μ*_F_ = 6.0 eV) for different laser field amplitudes. The plots show the eigenenergies and probability density distribution of the eigenstates along the junction. In each unit cell, the population of each eigenstate is divided into a contribution due to the Wannier functions that form the VB (blue) and CB (red). For a given *E*(*t*), current can only flow through the junction when the Wannier-Stark states at the terminal ends are properly energetically aligned with the lead’s chemical potential. Specifically, the terminal Wannier functions associated with the CB (or VB) need to be below (or above) the chemical potential for silica-metal charge exchange. The blue arrows indicate the flow of electrons. Only for *E* > 1.4 V Å^−1^ transport channels are open and significant current can be observed. **g** Currents entering the left (*I*_L_, black) and right (*I*_R_, red) contact during the photoinduced dynamics of the junction induced by *E*(*t*) with *φ* = *π* (in gray). The main plot shows the contribution due to the CB to the total current (electron transport) and the top-right inset that of the VB (hole transport). The energies and spatial probability distribution of the laser-dressed eigenorbitals for maximum and minimum laser amplitude are shown at the top-left and bottom-right corners, respectively. Net phase-controllable currents arise due to a difference in effective coupling between SiO_2_ and the metallic contacts for negative and positive field amplitude. In the CB, a burst of charge from the metal is observed around the central peak of the pulse for positive amplitude where charge injection is allowed. By contrast, for negative amplitudes transport through the CB is energetically blocked, leading to net charge transport

To understand the microscopic origin of the effect it is useful to interpret the quantum dynamics in terms of the instantaneous laser-dressed single-particle eigenstates of the silica and to examine how the energy of the laser-dressed levels match the chemical potentials of the contacts. The laser-dressed eigenstates are obtained by diagonalizing the Hamiltonian of the silica in the presence of the laser-matter interactions for a fixed electric field. Figure 8a–f shows the eigenenergies and probability density distribution of the eigenstates along the junction with the densities coarse-grained over unit cells. To enhance the interpretative value of the plots, the probability density of the eigenstates in each unit cell is divided into a contribution due to the Wannier states that form the VB (blue) and those that form the CB (red). The position of the chemical potential in the left and right contact, *μ*_L_ and *μ*_R_, are indicated by dashed lines and vary with the laser amplitude. The effect of the static electric field is to localize the silica eigenstates into so-called Wannier-Stark states. For the laser amplitudes in the experiment this localization is extreme, confining the eigenstates to 1–2 unit cells.

Since Stark effect dominate the dynamics and Zener tunneling does not play a significant role, in the absence of metallic contacts, the VB levels are occupied while those in the CB are empty during and after the laser pulse. Thus, for charge to flow between silica and contacts the Wannier-Stark states at the terminal ends of the junction need to be in proper energetic alignment with the contact’s Fermi sea. Specifically, for charge to flow from the VB into the contacts, the terminal VB Wannier-Stark states need to be above the contact’s chemical potential. Otherwise, hole transport is blocked. Similarly, for charge to flow from the contact into the CB the terminal CB Wannier-Stark states need to be below the chemical potential. Otherwise, electron transport is blocked. This basic energetic alignment picture is used below to develop an intuitive interpretation of the experiment.

### Origin of the threshold at $\left| {\boldsymbol{E}}_{0} \right|\sim \mathbf{1.4}$ V Å^−1^ to generate sizable currents

In both theory and experiments sizable currents require electric field amplitudes $\left| {E_0} \right| > 1.4$ V Å^−1^ (cf. Fig. 4a). To understand this threshold consider the laser-dressed eigenstates of the material and the level alignment shown in Fig. 8. For $\left| {E_0} \right|$ < 1.4 V Å^−1^ the CB levels at the terminal ends are above the chemical potential of the contacts and thus no charge can be transferred from the contacts into the CB. Similarly, the VB levels at the terminal ends are below the chemical potential of the contacts and charge transfer from the material into the leads is blocked by the Fermi sea. Thus, no significant currents are observed for $\left| {E_0} \right|$ < 1.4 V Å^−1^ because electron and hole transport are energetically blocked. At $\left| {E_0} \right|\sim 1.4$ V Å^−1^ this blocking of the charge transfer is removed and current starts to flow across the junction. For stronger fields $\left| {E_0} \right|\sim 1.7$ V Å^−1^ more levels satisfy the energy level alignment conditions leading to a larger currents.

### Mechanism for the generation of currents

To understand the origin of the currents, it is useful to focus on the separate electron and hole contributions to the total current. This can be done because the sum of these two individual contributions coincides with the full dynamics (Fig. 7). Figure 8g shows the current flowing through the CB under the influence of the laser pulse shown in gray ($\left| {E_0} \right| = 1.7$ V Å^−1^, *φ* = *π*). The current entering the right (left) contact is shown in red (black). An analogous diagram for the VB is shown in the top-right inset. The two additional insets show the laser-dressed single-particle eigenstates for the maximum positive *E* = 1.7 V Å^−1^ and negative *E* = −1.4 V Å^−1^ field amplitudes.

The net currents across the junction arise because of a difference in effective lead–silica couplings for positive and negative field amplitudes. To see this, consider transport through the CB first. For negative amplitudes, the field is not strong enough to push the terminal CB Wannier-Stark levels below the chemical potential of the contacts and little current is injected from the leads into the CB. By contrast, for a positive amplitude of *E* ~ 1.7 V Å^−1^ the electric field is strong enough to bring the right-end CB Wannier-Stark levels below *μ*_R_ leading to a large burst of charge being injected from the right contact into the leads. This imbalance between the effective coupling of the CB to the leads for positive and negative field amplitudes leads to a net electron current.

A similar situation occurs for hole transport (inset Fig. 8g). In this case, the field amplitude is strong enough to push the terminal Wannier-Stark levels above the chemical potential of the contact for the three central peaks, one positive and two negatives, of the pulse. For the other peaks the field is not strong enough to open significant channels for charge transport from the silica into the leads. This generates two bursts of charge injected into the right contact and one into the left one. The difference in effective lead-silica coupling for positive and negative field amplitude yields a net hole current along the device. The hole and electron current do not exactly cancel one another and give rise to a net current.

In this context, it becomes intuitively clear the origin of the phase control. When appreciable currents are observed ($\left| {E_0} \right| > 1.4$ V Å^−1^), the control maximum is achieved for *φ* ≈ 0, *π*. This is because this field maximizes the difference in laser intensity for negative and positive electric field amplitudes (Fig. 3b) and, thus, the difference in effective coupling between silica and right and left contacts. Since the identified mechanism is at play for a wide range of laser amplitudes, this is the origin of the insensitivity of *φ*^max^ on laser intensity identified in ref. ^20^. By contrast, when *φ* ≈ *π*/2 the field is antisymmetric with respect to time inversion around some time *t*′, i.e., *E*(*t* − *t*′) = −*E*(−(*t* − *t*′)) and the controllable current is small. This is because this field will have equal intensity for positive and negative field amplitude.

Further, we can now readily understand why the effect is independent of junction size. Due to Wannier-Stark localization only the 1–2 unit cells at the junction boundaries determine the charge dynamics. Thus by increasing the size of the junction one is not affecting the effectiveness of the scenario.

It is qualitatively useful to consider this control scenario in a minimal single-band model with *N* sites. In the presence of a static electric field of amplitude *E*(*t*), the energy of the Wannier-Stark states^25^ located at the terminal ends of the single-band material are $\epsilon _{\mathrm{R}} \approx \epsilon _0$ + $\frac{1}{2}eNdE(t)$ and $\epsilon _{\mathrm{L}} \approx \epsilon _0$ − $\frac{1}{2}eNdE(t)$, where $\epsilon _0$ corresponds to the onsite energies of the pristine material. In turn the chemical potentials of the right and left contacts are *μ*_R_ = *μ*_F_ + $\left( {\frac{{Nd}}{2} + u} \right)eE(t)$ and *μ*_L_ = *μ*_F_ − $\left( {\frac{{Nd}}{2} + u} \right)eE(t)$ where *Nd* + 2*u* is the total length of the junction and *u* is the distance of the lead-silica interface. In this model, the threshold for charge transport between the band and the metallic contacts occurs when $\epsilon _{\mathrm{R}}$ = *μ*_R_ or $\epsilon _{\mathrm{L}}$ = *μ*_L_. This yields a threshold electric field of $E_{\mathrm{L}}^ \ast$ = $\frac{{(\mu _{\mathrm{F}} - \epsilon _0)}}{{eu}}$ for the left contact and $E_{\mathrm{R}}^ \ast = - E_{\mathrm{L}}^ \ast$ for the right one. The key to achieve a current is then to use a field for which, for instance, the threshold $E_{\mathrm{L}}^ \star$ is contained in the pulse but $E_{\mathrm{R}}^ \star$ is not such that a net difference in effective lead-silica couplings for positive and negative field amplitudes emerges. To guarantee that the intensity $I\sim \left| E \right|^2$ is different for positive and negative amplitudes, *E*(*t*) cannot be antisymmetric with respect to time inversion around any given time. This can be achieved by using few-cycle pulses like the one employed in the experiment with *φ* ≠ (2*n* + 1)*π*/2 where *n* = 0, 1, 2, … or, alternatively, using two color *nω* + *mω* pulses where *n* is an even integer while *m* is odd.

This identified mechanism is reminiscent to the one proposed in ref. ^18^. While the qualitative ideas are identical an important technical difference, however, is that in the early study^18^ it was supposed that the chemical potentials of the contacts did not change with the laser field. This supposition leads to an effect that arises at much weaker fields. As shown in Fig. 4b–d (green line), when such a time dependence is not taken into account the simulations cannot recover the experimental observations.

## Discussion

We have presented atomistically detailed time-dependent quantum transport simulations of experiments^4,20^ that induce currents along metal-silica-metal nanoscale junctions using strong non-resonant few-cycle 4 fs laser pulses. The simulations are based on propagating the single-particle von Neumann equation for the junction using a state-of-the-art time-dependent non-equilibrium Green’s function method that, contrary to previous simulation and interpretational efforts, explicitly take into account the nanoscale nature of the experiment and the crucial role of the metallic contacts on the emergence of the effect. The simulations do not take into account possible effects of plasmons, screening or other processes that require feedback between the electromagnetic field and the material response.

Under these conditions, the simulations recover the experimental observations and offer an intuitive picture of the effect in which the temporal asymmetry of the incident radiation generates a difference in effective coupling of the silica to the left and right metallic contact and leads to a net phase-controllable current. Specifically, because the few cycle laser pulse in the experiment has different laser intensity for negative and positive field amplitude, through Stark shifts, such laser generates different metal-semiconductor band alignment for the left and right contacts leading to a net current across the nanojunction. Varying the carrier envelope phase controls the difference in intensity of the pulse for positive and negative field amplitudes and thus the sign and magnitude of the photoinduced currents. This identified mechanism is reminiscent to the early proposal in ref. ^18^.

An analysis of both simulation and experimental results suggest that previously proposed resonant 5 vs. 6 coherent control do not underlie the experimental observations. In addition, Wannier-Stark metallization and other possible mechanisms based on Zener tunneling do not underlie the simulated dynamics, and are thus not necessary for the emergence of the effect. Further, to explain the experimental observations it is not necessary to invoke mechanisms that involve the generation of virtual carriers. Additional progress in understanding the photoinduced dynamics in junctions requires experiments that address the relative importance of bulk and interfacial contributions^26^ and the length dependence of the effect at all relevant regimes of the laser-matter interaction.

Importantly, the simulations reveal that the experiment by Schiffrin et al.^4,20^ is based on Stark effects and not on near resonance multiphoton absorption. Thus, the experiment exemplifies the power of Stark-based strategies to control electronic properties and dynamics. These insights can be employed to interpret recent related experiments^27–30^ and to advance our ability to control electrons in matter using lasers.

## Methods

### Hamiltonian

The Hamiltonian for the composite metal-silica-metal junction is given by:

1 $$H(t) = H_{\mathrm{S}}(t) + H_{\mathrm{G}}(t) + H_{{\mathrm{SG}}},$$

where *H*_S_(*t*) describes the Hamiltonian of the silica, *H*_G_(*t*) the leads and *H*_SG_(*t*) the silica-lead couplings. The composite system is assumed to be well described by an effective single-particle Hamiltonian *H*(*t*) = $\mathop {\sum}\nolimits_{\nu \mu } {\kern 1pt} h_{\nu \mu }(t)c_\nu ^\dagger c_\mu$ where the operator $c_\nu ^\dagger$ (or *c*_*ν*_) creates (or annihilates) a fermion in a single-particle state *ν* and satisfies the usual fermionic anticommutation relations. As such, the electronic properties of the composite system are completely determined by the single-particle reduced density matrix *ρ*_*νμ*_(*t*) = $\left\langle {c_\nu ^\dagger c_\mu } \right\rangle$.

### Tight-binding model for laser-irradiated silica nanostructures

To obtain a first principle description of the silica and its interaction with a laser field, we computed the Bloch states and the band structure of bulk *α*-quartz and used that to construct an accurate generalized tight-binding model for the material and the transition dipoles required to capture the laser-matter interactions. Specifically, the ground-state band structure of *α*-SiO_2_^24^ was computed using DFT in the Vienna ab initio simulation package (VASP)^31^ with the modified Becke-Johnson (MBJ) meta-GGA functional^22^, and a plane-wave basis set with an energy cutoff of 650 eV. The calculated band gap of *α*-SiO_2_ is about 9 eV, in good agreement with experiment^32^. From the resulting Bloch eigenstates, an orthonormal basis of maximally localized Wannier functions (MLWFs) $\left\{ {\left| {\phi _n({\boldsymbol{r}},{\boldsymbol{R}}_l)} \right\rangle } \right\}$ was constructed via unitary transformation using Wannier90^23,33^. Here ***r*** is the electron coordinate and $\left\{ {\left| {\phi _n({\boldsymbol{r}},{\boldsymbol{R}}_l)} \right\rangle } \right\}$ is the *n*-th Wannier function localized on the *l*th unit cell associated with the real-space lattice vector ***R***_*l*_. The MLWFs were chosen to reproduce the band structure in the ([−8 eV, 14.5 eV]) energy window which includes 18 valence bands (VB) and 9 conduction bands (CB). The procedure resulted in *N*_*b*_ = 27 MLWFs per unit cell that quantitatively reproduce the band structure of *α*-SiO_2_ in a wide range of energies.

In dipole approximation, the Hamiltonian for the one-dimensional slab of *α*-SiO_2_ in the presence of a laser field polarized along the junction direction is given by

2 $$H_{\mathrm{S}}(t) = H_0 - \mu E(t),$$

where *H*_0_ is the Hamiltonian of the pristine silica, *μ* is the dipole operator and *E*(*t*) is the electric field of light. In the maximally localized Wannier basis, the Hamiltonian of *N* unit cells of *α*-SiO_2_ along a given crystallographic direction is given by

3 $$H_0 = \mathop {\sum}\limits_{l,l\prime = 1}^N {\kern 1pt} \mathop {\sum}\limits_{n,n\prime = 1}^{N_b} {\kern 1pt} h_{nl,n\prime l\prime }c_{nl}^\dagger c_{n\prime l\prime },$$

where $c_{n,l}^\dagger \left| 0 \right\rangle = \left| {\phi _n({\boldsymbol{r}},{\boldsymbol{R}}_l)} \right\rangle$ creates a fermion in MLWF $\left| {\phi _n({\boldsymbol{r}},{\boldsymbol{R}}_l)} \right\rangle$ and $\left| 0 \right\rangle$ is the vacuum state. Here $h_{nl,n\prime l\prime }$ = $\left\langle {\phi _n({\boldsymbol{r}},{\boldsymbol{R}}_l)} \right|H_0\left| {\phi _{n\prime }({\boldsymbol{r}},{\boldsymbol{R}}_{l\prime })} \right\rangle$ are the matrix element of the Hamiltonian among the Wannier states. In this microscopic model of the junction, the ${\boldsymbol{R}}_l = ld\widehat {\boldsymbol{d}}$ are chosen to be collinear and defined along a particular crystallographic direction $\widehat {\boldsymbol{d}}$ with lattice constant *d*. Since the $\left\{ {\left| {\phi _n({\boldsymbol{r}},{\boldsymbol{R}}_l)} \right\rangle } \right\}$ basis is maximally localized in real space (as shown in Fig. 1c) it suffices to only consider same cell *l* = *l*′ and nearest neighbor ($\left| {l - l\prime } \right| = 1$) contributions to the Hamiltonian. Hamiltonian matrix elements with unit cells that are located in directions perpendicular to $\widehat {\boldsymbol{d}}$ are neglected.

The total dipole operator *μ* = (*μ*_N_ + *μ*_*e*_) along the junction direction that determines the laser-matter interactions is also obtained from first-principle computations. Specifically, the electronic component of *μ* is given by

4 $$\mu _e = - e\mathop {\sum}\limits_{l,l\prime = 1}^N {\kern 1pt} \mathop {\sum}\limits_{n,n\prime = 1}^{N_b} \widehat {\boldsymbol{d}} \cdot {\boldsymbol{r}}_{nl,n\prime l\prime }c_{n,l}^\dagger c_{n\prime ,l\prime },$$

where *e* is the magnitude of the electronic charge, and ${\boldsymbol{r}}_{nl,n\prime l\prime }$ = $\left\langle {\phi _n({\boldsymbol{r}},{\boldsymbol{R}}_l)} \right|{\boldsymbol{r}}\left| {\phi _{n\prime }({\boldsymbol{r}},{\boldsymbol{R}}_{l\prime })} \right\rangle$ are the matrix elements of the position operator in the Wannier basis, which are computed with Wannier90. In turn, the nuclear dipole is constant throughout the simulation and given by *μ*_N_ = $e\mathop {\sum}\nolimits_{l = 1}^N {\kern 1pt} \mathop {\sum}\nolimits_{A \in l} {\kern 1pt} eZ_A\widehat {\boldsymbol{d}} \cdot {\boldsymbol{R}}_{A,l}$, where the second sum runs over all atoms *A* in cell *l* with position ***R***_*A*,*l*_ and atomic number *Z*_*A*_. The total junction length is taken to be *D* = *Nd* + *d* where *Nd* is the length of the silica and the extra *d* accounts for the approximate distance between the silica and the first layer of metallic atoms on each side of the junction.

### Metallic contacts and lead–silica interactions

The leads are described by *H*_G_(*t*) = $\mathop {\sum}\nolimits_{\alpha = {\mathrm{L}},{\mathrm{R}}} \mathop {\sum}\nolimits_q {\kern 1pt} \varepsilon _{\alpha q}c_{\alpha q}^\dagger c_{\alpha q}$ where $c_{\alpha q}^\dagger$ and *c*_*αq*_ are the fermionic operators for the lead states of energy *ε*_*αq*_ and *α* = L or R denotes the left or right contact, respectively. The leads are assumed to be in a state of thermal equilibrium with a density matrix described by the Fermi-Dirac distribution at temperature *T* = 300 K and with chemical potential *μ*_*α*_.

The leads and their interaction with the silica are described in the wide band limit (WBL). In this limit, the density of states in the metal is assumed to be constant and the Wannier functions that couple to the leads are supposed to couple identically to all lead levels. From a dynamical perspective, in WBL the metallic contacts behave as a Markovian reservoir that exchanges particles and energy with the material. As a model of the lead–silica interactions, we suppose that only the Wannier states $\left| {\phi _n({\boldsymbol{r}},{\boldsymbol{R}}_l)} \right\rangle$ in the terminal unit cells (*l* = 1 or *N*) couple to its adjacent contact. Further, the couplings to each lead is taken to be independent of the nature of the Wannier state. Thus, the silica-lead interaction is given by

5 $$H_{{\mathrm{SG}}} = \mathop {\sum}\limits_q {\kern 1pt} \mathop {\sum}\limits_{n = 1}^{N_b} \left( {{V_q^{\mathrm{L}}}c_{{\mathrm{L}}q}^\dagger c_{n1} + {V_q^{\mathrm{R}}}c_{{\mathrm{R}}q}^\dagger c_{nN} + {\mathrm{H.c.}}} \right),$$

where $V_q^\alpha$ is the coupling between level *q* in lead *α* and the Wannier states $\left| {\phi _n({\boldsymbol{r}},{\boldsymbol{R}}_l)} \right\rangle$ in the unit cell adjacent to it, and H.c. denotes Hermitian conjugate. The effective coupling between Wannier state $\left| {\phi _n({\boldsymbol{r}},{\boldsymbol{R}}_l)} \right\rangle$ and lead *α* is specified by the spectral density ${{\Gamma }}_\alpha (\varepsilon )$ = $2\pi \mathop {\sum}\nolimits_q \left| {V_q^\alpha } \right|^2\delta \left( {\varepsilon - \varepsilon _{\alpha q}} \right)$, a quantity that contains information about the characteristic frequencies of the leads and their coupling to the molecule. In the WBL the $V_q^\alpha$ and the leads’ density of states *ζ*^*α*^ = $\mathop {\sum}\nolimits_q {\kern 1pt} \delta \left( {\varepsilon - \varepsilon _{\alpha q}} \right)$ are assumed to be energy independent. In this case, the spectral density is also energy independent and given by

6 $${{\Gamma }}_\alpha = 2\pi \zeta ^\alpha \left| {V^\alpha } \right|^2.$$

In this work, we use *Γ*_L_ = *Γ*_R_ = *Γ* = 0.1 eV. The quantity *Γ* dictates the characteristic timescale, *ħ*/*Γ*, for charge exchange between silica and contacts and generates an effective Lorentzian broadening of the silica energy levels by 2*Γ*.

### Time-dependent transport

The time-dependent transport characteristics of the metal-silica-metal junction are characterized via the non-equilibrium Green’s function method (NEGF) as developed and implemented by Chen and colleagues ^21,34–37^. In TD-NEGF the current is obtained by solving the Liouville von Neumann equation for the single-particle electronic reduced density matrix in the presence of leads. In it, the current entering lead *α* is defined by *I*_*α*_(*t*) = −$e\frac{{\mathrm{d}}}{{{\mathrm{d}}t}}\left( {\mathop {\sum}\nolimits_q \left\langle {c_{\alpha q}^\dagger c_{\alpha q}} \right\rangle } \right)$ and the net current passing through the nanojunction is calculated as the average current flowing into the two leads *I*(*t*) = (*I*_L_(*t*) − *I*_R_(*t*))/2.

The employed method combines time-dependent density functional theory and NEGF^21,34–37^. Specifically, ref. ^35^ presents a computational efficient closed set of equations (Eqs. (3), (12) and (14) in ref. ^35^) to capture time-dependent transport by invoking the wide band limit and a Padé expansion of the Fermi distribution function. The former allows closing the resulting hierarchy of equations at first tier in the Hierarchical Equation of Motion sense. In turn, the Padé expansion allows for analytically solving the energy integrals that appear in the definition of the self-energies. Here, results were checked for convergence on the number of Padé functions (50) required to represent the leads, and on the integration time step (0.002 fs) of the Runge Kutta method of order four employed in the numerical integration of the equations of motion.

### Data availability

The data are available from the corresponding author upon reasonable request.

## Electronic supplementary material

Supplementary Information
