# Supplementary material for: Stark control of electrons along nanojunctions
Source: Nat Commun. 2018 May 25;9:2070. doi: 10.1038/s41467-018-04393-4 (PMC5970263; doi:10.1038/s41467-018-04393-4)
Supplement: Supplementary file 1 — Supplementary Information [file 41467_2018_4393_MOESM1_ESM.pdf]

Stark control of electrons along nanojunctions: Supplementary Information

Liping Chen,[1] Yu Zhang,[2,*] GuanHua Chen,[2,†] and Ignacio Franco[1,3,‡]

[1] *Department of Chemistry, University of Rochester, Rochester, New York 14627, USA*

[2] *Department of Chemistry, The University of Hong Kong, Pokfulam Road, Hong Kong*

[3] *Department of Physics, University of Rochester, Rochester, New York 14627, USA*

(Dated: April 24, 2018)

[*] Present address: Theoretical Division, Los Alamos National Laboratory, Los Alamos, New Mexico 87545, USA

[†] ghc@everest.hku.hk

[‡] ignacio.franco@rochester.edu

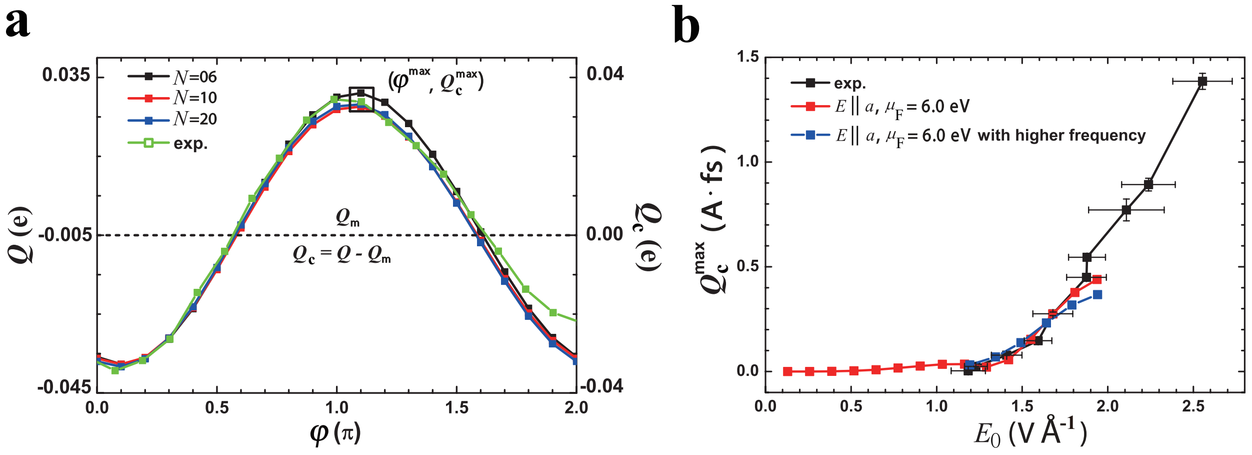

Supplementary Figure 1. **Influence of the band gap mismatch of the quasi one-dimensional silica nanowires on the laser control.** The one dimensional slabs of SiO_2 have a bandgap 16-18% larger than bulk because the tight-binding parameters along directions perpendicular to crystal growth are neglected. To test the influence of this feature of the model on the results, we performed simulations with a higher central frequency ω for the laser such that the experimental $E_{\mathrm{g}}/\hbar\omega = 5.3$ ratio is maintained. The plots contrast the resulting (a) phase control (maximum laser amplitude 1.7 V Å^{-1}, experiments are scaled) and (b) dependence of the maximum extracted charge $Q_{\mathrm{c}}^{\mathrm{max}}$ (simulations are scaled by the illumination cross-section η) against experiments and the simulations using a $\hbar\omega = 1.7$ eV ($E \parallel a$, $\mu_{\mathrm{F}} = 6.0$ eV). Experimental values are taken from Ref. [4]. Error bars denote standard deviation. Increasing the central frequency naturally changes the illumination cross section η (from 3.571×10^5 to 6.667×10^5 unit cells), but leaves the qualitative features of the control map unchanged.
